# Damage control surgery as a rescue procedure for ruptured hepatic subcapsular hematoma following ERCP for choledocholithiasis in a resource-limited setting: a case report

**DOI:** 10.1097/RC9.0000000000001123

**Published:** 2026-10-21

**Authors:** Mónica Waleska Flores Münchmeyer, Mildred Erminda Calel Anaya, Adolfo Antonio Borelly Acevedo Zelada

**Affiliations:** a Department of General Surgery, Hospital de Puerto Barrios, Instituto Guatemalteco de Seguridad Social, Puerto Barrios, Guatemala; b Department of Internal Medicine, Hospital de Puerto Barrios, Instituto Guatemalteco de Seguridad Social, Puerto Barrios, Guatemala

**Keywords:** case report, damage control surgery, emergency laparotomy, endoscopic retrograde cholangiopancreatography, hemoperitoneum, hepatic subcapsular hematoma

## Abstract

**Introduction:**

Hepatic subcapsular hematoma is a rare but potentially life-threatening complication of endoscopic retrograde cholangiopancreatography (ERCP). Rupture with hemorrhagic shock requiring staged damage-control surgery is exceptionally uncommon.

**Case presentation:**

A 23-year-old woman underwent ERCP with sphincterotomy, balloon extraction of a 10 mm common bile duct stone, and plastic biliary stent placement. After an approximately 6-hour interhospital transfer, she arrived with pallor, diaphoresis, dyspnea, nausea, vomiting, profound hypotension (60/30 mmHg), and tachycardia (125 beats per minute), but no abdominal pain. Hemoglobin decreased from 9.6 g/dL before ERCP to 6.6 g/dL before surgery. Computed tomography (CT) showed intraperitoneal fluid and extensive right hepatic subcapsular hemorrhagic changes without hollow viscus perforation or pancreatic inflammation. Emergency laparotomy revealed a ruptured right hepatic subcapsular hematoma and 900 mL of hemoperitoneum. Perihepatic packing and two staged re-explorations achieved definitive hemorrhage control.

**Clinical discussion:**

Transfer for angioembolization was unsafe because of ongoing shock, a minimum 6-hour journey, and the unavailability of interventional radiology on weekends. Damage control surgery provided rapid control of diffuse hepatic bleeding. The patient recovered without organ failure or delayed complications.

**Conclusion:**

Post-ERCP hepatic subcapsular hematoma should be considered in cases of unexplained anemia or hemodynamic instability, even without abdominal pain. CT should not delay definitive hemorrhage control. When embolization is unavailable or transfer is unsafe, perihepatic packing with planned re-exploration can be lifesaving.

## Introduction

Endoscopic retrograde cholangiopancreatography (ERCP) is an established therapeutic procedure for biliary and pancreatic disorders, but it carries risks, including pancreatitis, infection, bleeding, and perforation^[^1–3^]^. Hepatic subcapsular hematoma is a rare extraluminal hemorrhagic complication^[^4–8^]^ whose true incidence is unknown and may be underestimated because routine imaging is not performed after uncomplicated ERCP^[^9^]^.

HIGHLIGHTS

- A 23-year-old woman arrived in shock after a 6-hour interhospital transfer.
- Computed tomography revealed a ruptured hepatic subcapsular hematoma with hemoperitoneum.
- Severe hemorrhage occurred despite minimal abdominal pain.
- Perihepatic packing and staged re-explorations controlled the bleeding.
- Damage control surgery was lifesaving when embolization was unavailable.

Two mechanisms have been proposed: injury to peripheral intrahepatic biliary or vascular branches by the guidewire, and traction-related disruption of small vessels during balloon or basket extraction^[^8,9^]^. In this patient, cannulation and balloon extraction were reported as uncomplicated, so no specific traumatic maneuver could be identified.

By 2020, 61 cases had been summarized, including 14 ruptured hematomas^[^9^]^. Rupture was associated with higher reported mortality than non-rupture (21.4% vs 2.2%), and 78.6% of ruptured cases required surgery. Subsequent reports confirm that management must be individualized: stable, contained hematomas may be observed or treated with embolization or drainage, whereas rupture, peritoneal blood, or hemodynamic instability requires urgent invasive treatment^[^9,10^]^.

This report describes successful staged damage-control surgery for a ruptured post-ERCP hepatic subcapsular hematoma in a patient with profound shock and minimal abdominal symptoms at a regional hospital without interventional radiology. The educational value lies in the diagnostic clue of unexplained post-ERCP instability and in a practical operative rescue strategy when transfer is unsafe.

This case report has been prepared in accordance with the SCARE checklist^[^11^]^.

## Case presentation

A 23-year-old woman with a previous cesarean section for preeclampsia was admitted on 15 April 2026 with jaundice, fever, vomiting, and right-upper-quadrant pain. She had no additional comorbidities, denied anticoagulant use, had no history of coagulopathy, and was classified as American Society of Anesthesiologists physical status I. She received ampicillin/sulbactam, with resolution of fever, vomiting, and right-upper-quadrant pain before ERCP, although jaundice persisted. Ultrasonography on admission showed a 7 × 5 mm gallbladder fundus stone. Magnetic resonance cholangiopancreatography on April 22 showed a 7 × 6 mm distal common bile duct stone and an 8.6 mm gallbladder fundus stone.

ERCP was performed on 25 April 2026 at a facility in Chiquimula by a gastroenterologist with at least 10 years of specialist experience. Cholangiography demonstrated a 10 mm stone in the retroduodenal common bile duct (Fig. 1). Wide sphincterotomy and balloon sweeps achieved stone clearance, followed by placement of a 7 Fr × 10 cm plastic biliary stent. The endoscopist reported no difficult cannulation, multiple attempts, resistance, or other unusual events. Written informed consent for ERCP had been obtained after discussion of procedural risks.

**Figure 1.**
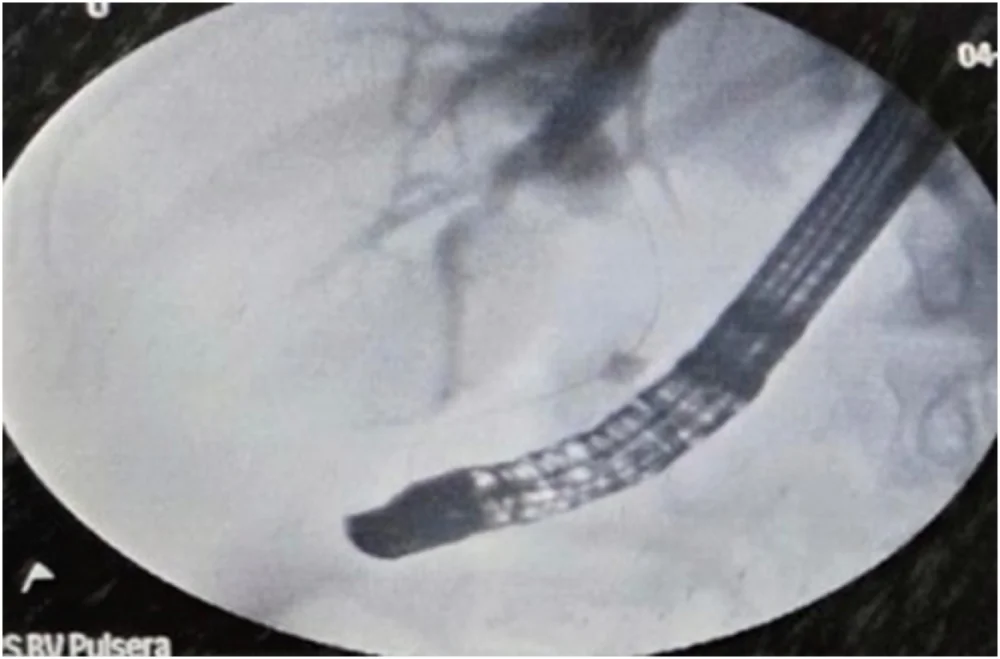
Fluoroscopic cholangiographic image obtained during endoscopic retrograde cholangiopancreatography.

After an interhospital transfer by road lasting approximately 6 hours, during which pallor and one episode of vomiting were observed, she returned to the referring hospital in Puerto Barrios. On arrival, she was pale and diaphoretic, with dyspnea and nausea, and no abdominal pain, distension, or altered mental status. She was taken to the emergency resuscitation bay with a blood pressure of 60/30 mmHg, a heart rate of 125 beats/minute, a respiratory rate of 22 breaths/minute, an oxygen saturation of 97%, and a temperature of 36.5 °C. She was transferred to the intensive care unit (ICU) for immediate resuscitation. Her hemoglobin had fallen from 9.6 g/dL before ERCP to 6.6 g/dL prior to surgery.

Resuscitation began with 2000 mL of Hartmann’s solution through peripheral venous access. Norepinephrine was prepared at a concentration of 16 mg in 250 mL of 5% dextrose, initiated at 10 mL/h and maintained at 8 mL/h during imaging and surgical decision-making. The patient and her husband were informed of the diagnosis, urgency, anticipated re-explorations, and operative risks; both authorized emergency surgery while the patient retained decision-making capacity.

Non-contrast computed tomography (CT) at approximately 19:25 on April 25 showed free intraperitoneal fluid and peripheral heterogeneous hepatic areas measuring 30–54 Hounsfield units, some subcapsular, consistent with post-procedural hemorrhage. There was no hollow viscus perforation. The pancreas had preserved morphology and density without peripancreatic inflammatory changes, and the bowel showed no wall thickening or obstruction. The biliary stent was in position (Figs 2A, B).

**Figure 2.**
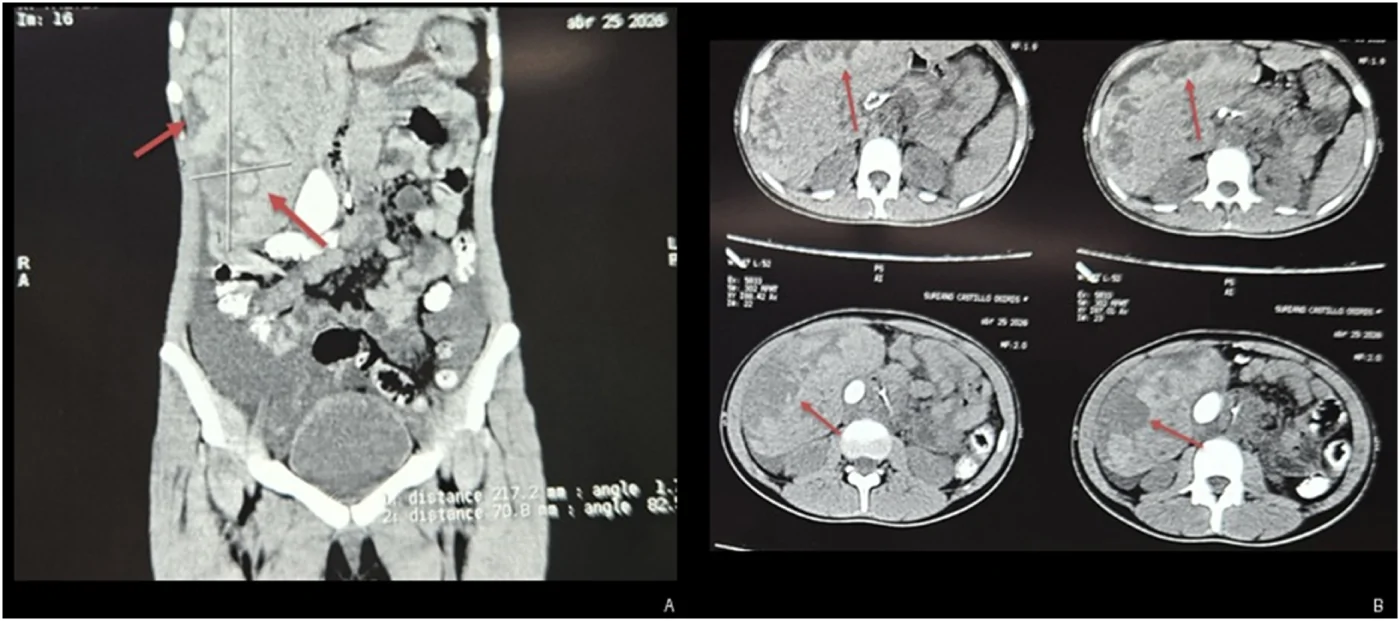
Computed tomography findings following ERCP. (A) Coronal reconstruction demonstrates an extensive right hepatic subcapsular hematoma. (B) Axial images demonstrate the right hepatic subcapsular hematoma and associated intraperitoneal fluid.

## Operative management

Transfer for angiography or embolization was not feasible: the patient was in profound shock, interventional radiology was unavailable within the hospital network over the weekend, and the only potential service was in Guatemala City – at least 6 hours away by road – with no assurance that a procedural team would be available.

Emergency exploratory laparotomy was therefore undertaken by the on-call general surgeon, who had 8 years of specialist experience, through a midline supra- and infraumbilical incision. Approximately 900 mL of hemoperitoneum and clots were evacuated. A large clot covered the anterior and lateral surfaces of the right hepatic lobe. Bleeding was diffuse and present over the hepatic surface, without a discrete point suitable for cautery or suture (Fig. 3A). Neither intraoperative ultrasonography nor Doppler was available. Seven laparotomy pads were placed for perihepatic packing. No drain was placed. The fascia was left open, and temporary abdominal closure was achieved by closing the skin with a continuous 1-0 nylon suture. Operative time was 80 minutes, and the total estimated blood loss was 1000 mL.

**Figure 3.**
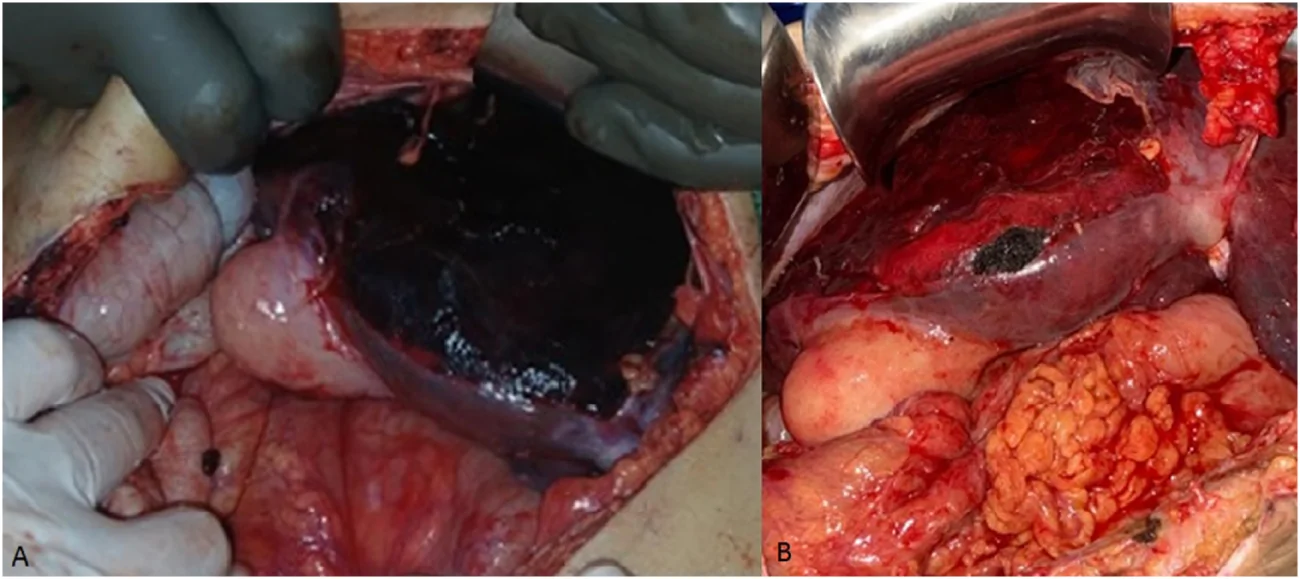
Intraoperative findings. (A) Large right hepatic subcapsular hematoma observed during the initial emergency laparotomy. (B) Operative field at the final re-exploration after removal of the perihepatic packing, showing no active bleeding.

The first re-exploration was performed at 16:00 on April 26 because of bleeding through the temporary abdominal closure. Through the same incision, 700 mL of hemoperitoneum and clots at the inferior border of the pack were evacuated. No focal active bleeding was identified. The liver was repacked with nine laparotomy pads, and the skin was again closed with continuous 1-0 nylon while the fascia remained open. Operative time was 65 minutes, and total estimated blood loss was 800 mL. A hepatobiliary surgeon in Guatemala City was consulted and recommended local stabilization with subsequent specialist follow-up.

At 12:45 on April 28, a second planned re-exploration was undertaken after restoration of hemodynamic stability, discontinuation of vasopressors, and stabilization of hemoglobin levels. Six hundred milliliters of blood were evacuated. All nine pads were removed, and no active bleeding was observed (Fig. 3B). Bilateral closed-suction Jackson-Pratt drains were placed. The fascia was closed with continuous 1-0 polyglactin, and the skin with surgical staples. Operative time was 95 minutes, and total estimated blood loss was 650 mL. No further packing was left in situ.

Three units of packed red blood cells and 1 unit of fresh frozen plasma were given during the initial operation and immediate ICU period; four additional units of packed red blood cells and 3 units of fresh frozen plasma were administered over the subsequent 48 hours, which included the first re-exploration. Calcium and vitamin K were administered. Tranexamic acid was unavailable. Platelets and cryoprecipitate were not required, and coagulation tests remained normal. Norepinephrine was discontinued approximately 24 hours after the initial operation.

## Postoperative course and follow-up

The patient remained intubated and mechanically ventilated through both re-explorations and was extubated 48 hours after definitive closure. Empiric intravenous vancomycin (1 g every 12 hours) and meropenem (1 g every 8 hours) were administered for 10 days; no cultures were obtained. Enoxaparin 40 mg subcutaneously every 24 hours was initiated 48 hours after definitive surgery and discontinued after ICU discharge when adequate mobilization was achieved.

The combined output of both Jackson–Pratt drains was approximately 50 mL during the first 48 hours, with progressive daily reduction. The left drain was removed 5 days after placement, and the right drain after 7 days, when output ceased. The patient left the ICU on May 6 and was discharged on 13 May 2026 without organ failure, wound complications, infection, recurrent bleeding, or other postoperative adverse events. No postoperative CT or ultrasonography was available.

On May 12, clinical recovery and substantial improvement in laboratory parameters were documented (Table 1). At follow-up on June 15, she remained asymptomatic without recurrent bleeding or delayed complications. She initially declined transfer for hepatobiliary follow-up. Stent removal remains planned as recommended by the hepatobiliary surgery department but is awaiting scheduling. Elective cholecystectomy has not yet been performed.

**Table 1:** Serial laboratory evolution.

Parameter	Pre-ERCP	Post-ERCP/pre-op	Post initial operation^a^	Hospital discharge
Hemoglobin (g/dL)	9.6	6.6	5.8	11.3
WBC (×10^3^/µL)	8.9	28.59	24.31	10.45
Neutrophils (%)	59.3	86.6	83.6	65
Platelets (×10^3^/µL)	277	398	307	485
Total bilirubin (mg/dL)	9.16	7.45	7.2	1.26
AST (U/L)	152	167	188	16.6
ALT (U/L)	210.8	159.6	162	12.1
ALP (U/L)	692.8	756	701	99.6
GGT (U/L)	449.6	255	304	37.5
Lactate (mmol/L)	–	–	1.37	0.9
Amylase (U/L)	111	121	136	114
Lipase (U/L)	79	78	85	101

^a^ Immediately after the initial operation, before completion of transfusion.

## Discussion

Hepatic subcapsular hematoma should be considered in the differential diagnosis when new-onset anemia, hypotension, tachycardia, pallor, dyspnea, fever, or right-upper-quadrant symptoms occur. Abdominal pain is common in published cases, but its absence does not exclude catastrophic bleeding^[^9–12^]^. In this patient, profound shock with minimal abdominal symptoms made the decline in hemoglobin and circulatory findings more informative than pain. Cross-sectional imaging established hepatic hemorrhage and excluded perforation and pancreatic inflammatory changes, permitting focused operative decision-making.

Management depends on hemodynamic status, rupture, ongoing blood loss, infection, and local resources^[^10,12^]^. Stable contained hematomas have been managed with observation, transfusion, antibiotics, embolization, or percutaneous drainage^[^13–15^]^.

Published surgically managed cases illustrate distinct indications and outcomes^[^9,12,16^]^. The present case is not the first report of damage control surgery for this complication^[^16^]^. This case contributes an example of survival without organ failure after immediate perihepatic packing, repeated assessment, and definitive closure in a regional hospital where embolization was unavailable and transfer was unsafe. Diffuse bleeding without a target for cautery or suture made packing the least destructive, rapid method of hemorrhage control, consistent with damage control principles used for severe hepatic bleeding^[^9,16^]^.

Counseling must accompany both phases of care. Before ERCP, the expected benefits and recognized complications should be discussed, and written consent should be obtained. When an unexpected hemorrhagic complication occurs, the patient and family should receive prompt, comprehensible information about the severity, alternatives, transfer feasibility, emergency operative treatment, and the possibility of re-exploration. This approach was followed in the present case.

## Conclusion

A ruptured hepatic subcapsular hematoma should be considered after ERCP when anemia or hemodynamic instability develops, even if abdominal pain is absent.

- In this case, CT demonstrated hepatic hemorrhage and excluded perforation or pancreatitis; however, imaging and transfer should not delay hemorrhage control in profound shock.

- When embolization is unavailable or transfer is unsafe, perihepatic packing with physiologic resuscitation and planned re-exploration may provide a lifesaving strategy.

## Data Availability

The anonymized data supporting the findings of this case report are available from the corresponding author upon reasonable request, subject to patient confidentiality and applicable institutional requirements.

## Patient perspective

The patient expressed gratitude for her recovery but reported persistent anxiety regarding subsequent procedures.
